# Supplementary material for: Active site specificity profiling datasets of matrix metalloproteinases (MMPs) 1, 2, 3, 7, 8, 9, 12, 13 and 14
Source: Data Brief. 2016 Feb 22;7:299–310. doi: 10.1016/j.dib.2016.02.036 (PMC4777984; doi:10.1016/j.dib.2016.02.036)
Supplement: Supplementary file 10 — Supplementary material [file mmc10.zip › WebPICS_hMMP3_G_1%/P2.html]

 

PICS results


|  |  |
| --- | --- |
| **P2\_A**  29 in 151 sites   19.2 %    effects > 10 perc. pnts.  (vice-versa in brackets)  P3\_V: 17.9 (20.8)   P1prime\_I: -10.3 (-11.5) |  |
| **P2\_C**  7 in 151 sites   4.6 %    effects > 10 perc. pnts.  (vice-versa in brackets)  P3\_L: 31.6 (13.0)   P1prime\_I: 39.9 (10.8)   P2prime\_C: 26.6 (62.1)   P2prime\_H: 39.6 (55.4) |  |
| **P2\_F**  6 in 151 sites   4.0 %    effects > 10 perc. pnts.  (vice-versa in brackets)  P1\_Q: 24.7 (11.4)   P1prime\_Q: 26.7 (16.0)   P1prime\_V: 52.8 (15.0)   P2prime\_I: 38.1 (12.7)   P3prime\_I: 25.4 (12.7)   P3prime\_N: 42.7 (23.3) |  |
| **P2\_G**  13 in 151 sites   8.6 %    effects > 10 perc. pnts.  (vice-versa in brackets)  P1\_K: 17.1 (24.7)   P3prime\_A: 21.3 (10.6) |  |
| **P2\_H**  6 in 151 sites   4.0 %    effects > 10 perc. pnts.  (vice-versa in brackets)  P3\_I: 24.7 (11.4)   P1\_Y: 12.1 (10.3)   P1prime\_Y: 13.4 (16.0)   P2prime\_R: 41.4 (19.1)   P2prime\_T: 26.7 (16.0) |  |
| **P2\_L**  17 in 151 sites   11.3 %    effects > 10 perc. pnts.  (vice-versa in brackets)  P3\_A: 16.2 (13.7)   P3\_I: 26.7 (34.9)   P1\_N: 19.4 (13.7)   P1prime\_C: 16.9 (28.7)   P2prime\_I: 17.5 (16.5)   P2prime\_T: 16.9 (28.7)   P3prime\_L: 12.2 (12.2) |  |
| **P2\_Q**  14 in 151 sites   9.3 %    effects > 10 perc. pnts.  (vice-versa in brackets)  P3\_P: 35.8 (11.6)   P1\_A: 11.5 (10.7)   P1\_Y: 16.8 (33.6) |  |
| **P2\_S**  13 in 151 sites   8.6 %    effects > 10 perc. pnts.  (vice-versa in brackets)  P3\_L: 19.5 (14.9)   P1\_H: 12.1 (31.4)   P2prime\_K: 22.6 (12.2) |  |
